# Impact of Atmospheric Conditions and Source Identification of Gaseous Polycyclic Aromatic Hydrocarbons (PAHs) during a Smoke Haze Period in Upper Southeast Asia

**DOI:** 10.3390/toxics11120990

**Published:** 2023-12-05

**Authors:** Wittaya Tala, Pavidarin Kraisitnitikul, Somporn Chantara

**Affiliations:** 1 Environmental Science Research Center (ESRC), Faculty of Science, Chiang Mai University, Chiang Mai 50200, Thailand; pavidarin.k@cmu.ac.th (P.K.); somporn.chantara@cmu.ac.th (S.C.); 2 Office of Research Administration, Chiang Mai University, Chiang Mai 50200, Thailand; 3 Environmental Chemistry Research Laboratory (ECRL), Department of Chemistry, Faculty of Science, Chiang Mai University, Chiang Mai 50200, Thailand

**Keywords:** air pollution, diagnostic ratios, gaseous PAHs, meteorological conditions, northern Thailand

## Abstract

Gaseous polycyclic aromatic hydrocarbons were measured in northern Thailand. No previous studies have provided data on gaseous PAHs until now, so this study determined the gaseous PAHs during two sampling periods for comparison, and then they were used to assess the correlation with meteorological conditions, other pollutants, and their sources. The total concentrations of 8-PAHs (i.e., NAP, ACY, ACE, FLU, PHE, ANT, FLA, and PYR) were 125 ± 22 ng m^−3^ and 111 ± 21 ng m^−3^, with NAP being the most pronounced at 67 ± 18 ng m^−3^ and 56 ± 17 ng m^−3^, for morning and afternoon, respectively. High temperatures increase the concentrations of four-ring PAHs, whereas humidity and pressure increase the concentrations of two- and three-ring PAHs. Moreover, gaseous PAHs were estimated to contain more toxic derivatives such as nitro-PAH, which ranged from 0.02 ng m^−3^ (8-Nitrofluoranthene) to 10.46 ng m^−3^ (1-Nitronaphthalene). Therefore, they could be one of the causes of local people’s health problems that have not been reported previously. Strong correlations of gaseous PAHs with ozone indicated that photochemical oxidation influenced four-ring PAHs. According to the Pearson correlation, diagnostic ratios, and principal component analysis, mixed sources including coal combustion, biomass burning, and vehicle emissions were the main sources of these pollutants.

## 1. Introduction

Polycyclic aromatic hydrocarbons (PAHs) are organic molecules containing only carbon, which have hydrogen atoms and two or more fused aromatic rings in a variety of structural configurations [1]. They are an important indicator of air pollution in ambient air since they are the most widely dispersed class of human carcinogens with mutagenic characteristics [2,3]. Furthermore, at low concentrations, they are responsive to humans and might combine synergistically with other air pollutants to cause serious health problems. As a result, several of them have been listed on the lists of national and international health agencies [4]. Although PAHs are commonly produced by biogenesis (e.g., volcanic eruptions, plant synthesis, vegetative decay, and rare minerals), most of them are produced by human activities (e.g., incomplete combustion of fossil fuels or carbon-containing organic compounds, industrial processes, and biomass burning) [5]. They have a proclivity to spread between the gaseous and particulate phases in the ambient air. This partitioning is caused by their chemical properties as well as the meteorological conditions (temperature and relative humidity) [6]. Several studies found two- to three-ring PAHs are a gaseous phase, while five- to seven-ring PAHs are a particulate phase. However, both phases are found in four-ring PAHs [7,8,9]. Although gaseous PAHs are less carcinogenic/mutagenic, there are many in the atmosphere and they can interact with other pollutants to produce derivatives through processes such as sulfonation, nitration, and photo-oxidation [10,11,12]. These are more dangerous than their parent PAHs because of their direct-acting mutagenicity and carcinogenicity, as well as a significant potential for toxicity at low concentrations [13,14]. For example, 1,3-dinitropyrene (1,3-DNP) and 1,8-dinitropyrene (1,8-DNP) are 6.3 × 10^4^ and 1.1 × 10^5^ times more mutagenic than benzo [a] pyrene, respectively [15]. Gaseous PAHs have also been shown to have more source-specific properties than particle-phase PAHs [16,17,18]. Because the dynamic range for ambient air reactivity is rather short, ranging from seconds to days [16,19], the data obtained demonstrated that gaseous PAHs are more prone to numerous reactions than particulate PAHs [20]. As a result, a long sampling period of gaseous PAHs (i.e., 24 h and 48 h) [19], similar to the particulate-phase sampling period (i.e., PM_2.5_) or a combination with the particulate phase [21], may provide an underestimation of gaseous PAHs. The loss of important data can also lead to bias in the quantification of health risk assessment. As a result, short-term air sampling may provide suitable information for characterizing gaseous PAHs and improving the knowledge of behavior, fate, and circumstances that may encourage further research in chemicals such as PAH derivatives. It is still unknown how meteorological variables affect the concentration and variation of gaseous PAHs in upper Southeast Asia (e.g., Thailand, Cambodia, Laos, Vietnam, and Myanmar), where air pollutants have frequently been studied as the most common particle pollutant from Thailand and its upper Southeast Asian neighbors which are agriculture-based countries, resulting in the production of a large number of agricultural residues, which are frequently burned in the field or used by agro-industries (outfield) to generate energy and electricity. Biomass burning is also common in the area to prepare for the next crop cycle and to remove weeds, insects, and animals. Additionally, emissions from forest fires are a significant source of air pollution. These emissions have been linked to a variety of causes, including drought, prevailing wind patterns, and intentional fires. Furthermore, because Chiang Mai is one of the most developed areas in Thailand, it has experienced rapid urbanization over the last 20 years, resulting in a significant impact on the environment, particularly on the region’s air quality. Therefore, the objectives of this study were to determine (1) the concentration of gaseous PAHs in northern Thailand (2), the role of meteorological conditions affecting the variability of gaseous PAH congeners, and (3) the potential sources of gaseous PAHs in this area. The results from this study will fill a data gap, and this is crucially important for a better understanding of the changes in gaseous PAH composition and distribution in upper Southeast Asia (U-SEA) and the potential of gaseous pollutants in this area.

## 2. Materials and Methods

### 2.1. Sampling Site and Sample Collection

**Figure 1:**
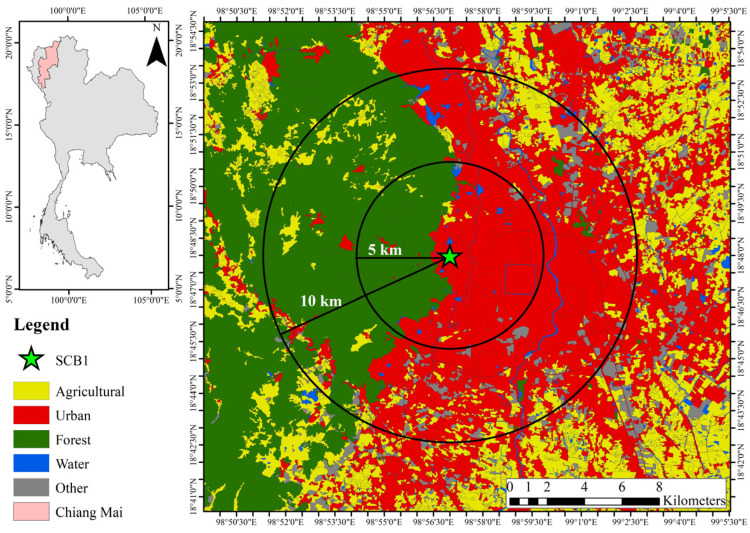
Geographical location of the study.

The gaseous PAH samples were collected from ambient air on the rooftop of the nine-story Science Complex Building 1 (SCB1), Chiang Mai University, at a height of 373 m above the mean sea level (latitude 18°48′5.13″ N and longitude 98°57′12.16″ E) between 24 April and 8 May 2015. Two sampling periods were held on the fifteenth day, in the morning and in the afternoon, to evaluate the variations in the gaseous PAHs. Every sampling period involved the collection of air samples at the breathing zone (about 1.5 m) over a period of two hours. Thirty samples in total were collected. This sampling period was chosen because there are high levels of PM_2.5_ from biomass burning in U-SEA each year. Furthermore, during this time, Chiang Mai has been named the world’s most polluted city, so it is reasonable to assume that high levels of other types of air pollutants were presented in the same trend, such as gaseous PAHs [22]. Gaseous PAHs have never been studied in this area until now. Therefore, the data obtained show gaseous PAHs in the ambient air during the smoke haze period. The results could be used to calculate the PAHs’ derivatives, which are more toxic. This sampling site was selected for gaseous PAHs from the ambient air at the receptor site as shown in Figure 1.

Prior to sampling, all the solid sorbents were cleaned up using lab-made hot extraction equipment for 6 h in dichloromethane (DCM) to remove the PAH contaminants until the total PAHs were identified as having less than 5 μg per gram of solid sorbent in accordance with Spicer et al. [23]. They were then dried in the oven before being stored in sealed desiccators. A combination of sampling tubes containing 250 mg and 150 mg were employed to collect gaseous PAHs during the duration of the sampling period, with an air flow rate of 4 L min^−1^ and a vertical position of 1.5–2.0 m above the ground.

### 2.2. Chemicals and Standards

The standard reference solution was supplied by Restex (Bellefonte, PA, USA), with additional components as follows: naphthalene (NAP), acenaphthylene (ACY), acenaphthene (ACE), fluorene (FLU), phenanthrene (PHE), anthracene (ANT), fluoranthene (FLA), pyrene (PYR), benzo[a]anthracene (BaA), chrysene (CHR), benzo[b]fluoranthene (BbF), benzo[k]fluoranthene (BkF), benzo[a]pyrene (BaP), dibenz[a,h]anthracene (DbA), indeno [1,2,3-cd]pyrene (IND), and benzo[g,h,i]perylene (BPER). The deuterated internal standards acenaphthene-d10 (ACE-d10) were obtained from Supelco (Mainz, Germany). All organic solvents used in this research were of high-performance liquid chromatography (HPLC)-grade purity and were obtained from RCI Labscan (Bangkok, Thailand). All stock solutions were prepared in a mixture of hexane/dichloromethane (1/1) to include the mixed PAH stock standards (20 µg mL^−1^) and the deuterated internal standard (50 µg mL^−1^). Stock solutions were then stored in amber bottles at −20 °C to avoid photo-degradation.

### 2.3. Sample Preparation

The extraction of 8-PAHs was carried out in accordance with Tala and Chantara [24]. Briefly, the 8-PAHs adsorbed on a solid sorbent were extracted via ultrasonic extraction with DCM/2-pro (4/1, *v*/*v* at a low temperature (≤10 °C)) for 30 min (Elma, WA, USA). After that, the extracts were filtered using disposable syringe filters (Nylon, 25 mm, 0.45 μm, Minipore, Burlington, MA, USA). They were then concentrated to 0.800–1.000 mL using rotary evaporation (Heidolph, Schwabach, Germany) before the solvent was changed to 2-pro/H_2_O (1/1, *v*/*v*) which produced a final volume of 2 mL. Following Tala and Chantara [25], the reconstituted solution was cleaned further. These solutions were cleaned using a commercial SPE cartridge containing 200 mg bonded silica absorbents. Prior to adding sodium sulfate anhydrous to the effluent, the 8-PAHs were eluted with 3 mL of Hex/DCM/2-pro (1/1/0.1). Then, the effluent was concentrated to around 800 mL using a rotary evaporator once again. Finally, ACE-d8 was added as an internal standard before the eluent was used to dilute the solutions to 2 mL for the detection of 8-PAHs, namely two-ring PAHs (NAP), three-ring PAHs (ACY, ACE, FLU, PHE, FLA), and four-ring PAHs (PHE and PYR). The efficiency of the sample treatment was investigated by referring to the recovery values of 8-PAHs using the spiking method and SRM 1649b. The spiking method recoveries ranged from 81% (NAP) to 96% (ANT), while the SRM 1649b recoveries were 64% (NAP)–98% (ACY and FLU) (Table S1, Supplementary Material).

### 2.4. Instrumental Analysis

The chromatographic analysis was carried out in a 7820A gas chromatograph (GC) equipped with a splitless injector and a 5977E mass spectrometer (MS) (Agilent Technologies, Santa Clara, CA, USA). Ultra-pure helium (He) gas was used as a carrier gas at a flow rate of 1.0 mL min^−1^. The temperatures of the injector, transfer line, and detector were 300 °C, 230 °C, and 150 °C, respectively. Separation was accomplished with a HP5-MS-UI fused silica capillary column (30 m length × 0.25 mm i.d. × 0.25 μm film thickness, Agilent J&W GC column) for 8-PAHs. The splitless injection of a 1 μL sample was performed with a 6 min solvent delay time to avoid the saturation of the mass spectrometer detector. The GC oven temperature was initially set at 70 °C and held for 2 min, then ramped up to 240 °C (8 °C min^−1^), held for 2 min, and then further ramped up to 260 °C (10 °C min ^−1^). After being held at 260 °C for 6 min, the oven was ramped up to 285 °C (15 °C min^−1^). Finally, it was held for 5 min to achieve a running time of less than 45 min. The MS was operated in the electron ionization (EI) mode at 70 eV. The temperatures of the injector, transfer line, and detector were 300 °C, 230 °C, and 150 °C, respectively. The sample was injected at a volume of 1 μL using the splitless mode on the column. A mass range of *m*/*z* 50–350 was recorded in full scan mode to identify each of the PAHs. The selective ion monitoring (SIM) mode was used for both qualitative and quantitative analyses. The identification of 8-PAHs was based on a match with the retention times and the ion ratios of the target quantification ions.

### 2.5. Quality Control

The LOD (limit of detection) and LOQ (limit of quantification) were tested via 10 injections of the lowest concentration (1 ng mL^−1^) of the mixed PAH standards. The results demonstrated that the LOD of 8-PAHs ranged between 0.030 and 0.053 ng mL^−1^, while the LOQ ranged between 0.101 and 0.177 ng mL^−1^.

The precision of the method was obtained from ten injections of low and high concentrations (6 and 60 ng mL^−1^) into the GC-MS. These values are presented in terms of the relative standard deviation (% RSD). Repeatability ranged between 0.57 and 1.48% RSD (6 ng mL^−1^), and the values ranged from 0.46 to 3.49% RSD (60 ng mL^−1^). The reproducibility was between 3.70 and 7.52% for 6 ng mL^−1^ and between 3.60 and 9.67% for 60 ng mL^−1^.

The linearity of the method was obtained from the results (signal) which were directly proportional to the concentration of individual PAHs within a given range. Excellent calibration graph values with a correlation coefficient (r^2^) of >0.995 ranged from 1 to 3000 ng mL^−1^ for all 8-PAHs. All the satisfaction data are presented in Table S2 (Supplementary Material), as well as the assessment of the gaseous PAHs in the ambient air.

To determine the efficiency of the 8-PAH determination from ambient air, all compounds were spiked with known amounts onto a solid sorbent prior to sampling. Both spiked and non-spiked solid sorbents were subjected to the same procedure for analysis via GC-MS. The following recoveries were obtained as follows: NAP (55%), ACY (65%), ACE (74%), FLU (86%), PHE (90%), ANT (87%), FLA (102%), and PYR (93%) (Table S3, Supplementary Material).

### 2.6. Air Pollution Data

We obtained the hourly average pollution data from the Pollution Control Department (PCD) [22] in northern Thailand at (35t) Chiang Mai City Hall Air quality station and (36t) Yupparaj Wittayalai School Air quality station in Chiang Mai Province. Individual data were obtained, including meteorological conditions (wind speed, net radiation, temperature, pressure, and relative humidity) and other pollutants (nitrogen dioxide (NO_2_), nitric oxide (NO), nitrogen oxides (NOX), carbon monoxide (CO), sulfur dioxide (SO_2_), ozone (O_3_), and particulate matter (PM_2.5_ and PM_10_).

### 2.7. Statistical Analysis

All the data were collected and analyzed using the SPSS statistical software 29.0.10 package to report on the association between 8-PAHs and meteorological conditions. All statistical inferences were conducted at a 5% alpha level.

## 3. Results and Discussion

### 3.1. Preliminary Measurement

**Figure 2:**
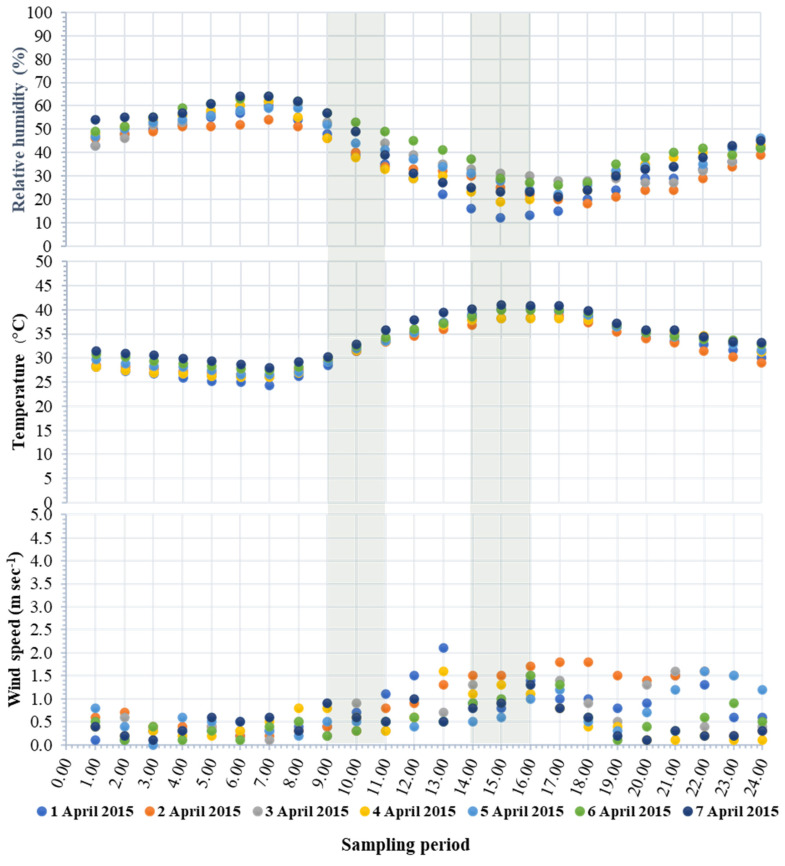
Hourly variations in meteorological conditions during the sampling period of gaseous PAHs from the ambient air.

Due to the physico-chemical mechanism of gaseous pollutants during air movement (i.e., photolysis, thermal degradation), the sampling duration is one of the most important factors which influences the measurement of gaseous PAHs in the ambient air [26,27,28]. For example, Ojeda-Castillo et al. [19] found that decreasing the sampling period by four times increased the detection of gaseous PAHs by 1.70–3.17 times, whereas Eiguren-Fernandez [8] showed that decreasing the sampling period by seven times increased the NAP concentration by more than 2.6 times. Therefore, it is important to emphasize that long sampling periods as in particle-phase (i.e., PM_2.5_) collection might result in an underestimation of gaseous PAHs. Furthermore, accurate data collection is crucial, especially in urban areas as in Chiang Mai province, where pollutants are known to come directly from a wide variety of sources during the dry season, such as from fuel combustion and biomass burning [29]; therefore, short sampling periods might reduce the matrix effect in ambient air. During this period, PM_2.5_, an important pollutant in this area, was found to be at a high level of concentration. During the selected sampling time, the hourly value of PM_2.5_ was in the range of 87 ± 27 μg m^−3^ (Min = 17 μg m^−3^ to Max = 167 μg m^−3^). For all the 168 values, it was found that 95% exceeded the standard values (37.5 µg m^−3^ 24 h average) [22]. Meteorological factors including relative humidity, temperature, and wind speed are normally important fluctuations within a day, and their values were considered [30,31,32,33] before the sampling time and sampling duration were selected, as shown in Figure 2. Each parameter provided a distinctive dynamic pattern. However, each parameter showed the same trend throughout the real-time monitoring. Based on these results, two time intervals (9–11 am and 2–4 pm) within a day were selected to represent the short-term diurnal fluctuation of gaseous PAHs.

### 3.2. Characteristics of Gaseous PAH Concentrations in the Ambient Air

Table 1 shows the average concentrations of individual PAHs, as well as the standard deviation (SD) and minimum-maximum concentrations detected during this study. The average concentrations were found to be 62 ±19 ng m^−3^ (NAP), 4.1 ± 3.5 ng m^−3^ (ACY), 6.9 ± 1.1 ng m^−3^ (ACE), 11 ± 2.7 ng m^−3^ (FLU), 20 ± 2.8 ng m^−3^ (PHE), 2.8 ± 0.6 ng m^−3^ (ANT), 5.6 ± 1.5 ng m^−3^ (FLA), and 5.8 ± 1.7 ng m^−3^ (PYR). NAP, PHE, FLU, ACE, PYR, FLA, ACY, and ANT are listed in decreasing order of concentration. Furthermore, two-ring PAHs (62 ± 19 ng m^−3^) were found to be the most prevalent, followed by three-ring PAHs (45 ± 6.3 ng m^−3^) and four-ring PAHs (11 ± 3.0 ng m^−3^), respectively.

**Figure 3:**
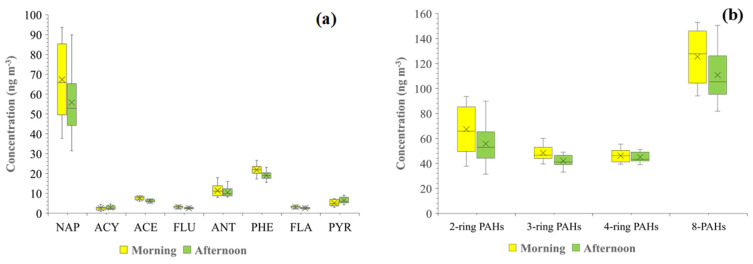
Box and whisker plots for gaseous PAHs in the ambient air based on (**a**) individual PAHs and (**b**) the number of rings between morning and afternoon periods. In this figure, mean values are indicated by the black crosses, central horizontal bars are the medians, and the lower and upper limits in the boxes are the 25th and 75th percentiles, respectively. The error bars show the range (lowest to highest) obtained.

Additionally, this study compared the morning and afternoon periods, and the results are shown as box plots in Figure 3. Except for ACY and PYR, the results showed that some gaseous PAHs were significantly different from one another after we conducted a pair test, and most of the gaseous PAH levels were found to be higher in the morning (i.e., NAP, ACE, FLU, ANT, PHE, and FLA). The range of gaseous PAH concentrations in the atmosphere in the morning and afternoon was 0.88 ng m^−3^ (ACY) to 94 ng m^−3^ (NAP) and 1.87 ng m^−3^ (ACY) to 90 ng m^−3^ (NAP), respectively. In terms of the number of rings, the morning had a higher concentration than the afternoon for all rings. The concentration ranged from 46 ng m^−3^ (four-ring PAHs) to 67 ng m^−3^ (two-ring PAHs) for the morning period and 42 ng m^−3^ (three-ring PAHs) to 56 ng m^−3^ (two-ring PAHs) for the afternoon.

**Figure 4:**
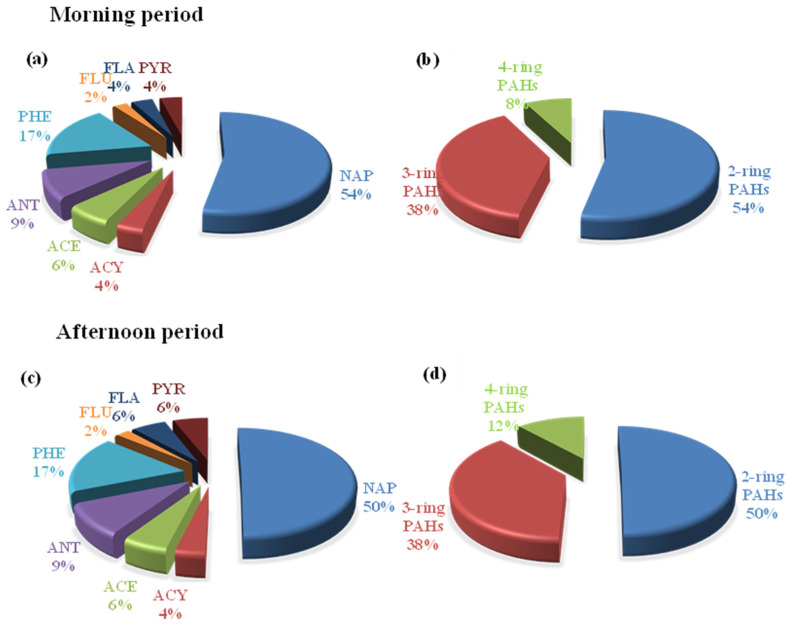
Contribution of gaseous PAHs during the morning period, for (**a**) individual PAHs and grouped by the (**b**) number of rings, and in the afternoon period, for (**c**) individual PAHs and grouped by the (**d**) number of rings.

The dominant compounds in both sampling periods were in the same order in terms of their contributions. NAP, PHE, and ANT were 54%, 17%, and 9.1%, respectively, in the morning period, whereas those compounds were 50%, 17%, and 9% in the afternoon period (Figure 4a,c). Furthermore, when the number of rings was compared, it was shown that the highest contributor was found in the two-ring PAHs (54 and 50%), followed by the three-ring PAHs (38 and 38%) and the four-ring PAHs (8 and 12%), respectively (Figure 4b,d).

Due to the variability in gaseous PAH concentrations between morning and afternoon, the SPSS statistical approach was chosen to calculate the precise differences between the morning and afternoon periods. While investigating individual compounds in greater depth, it was found that the levels of ACE, FLU, PHE, FLA, and PYR were significantly higher in the morning period. Therefore, this could be because they show that ACE, FLU, PHE, FLA, and PYR might be more affected by the atmospheric conditions. Moreover, the number of rings were compared; we can see that the levels of three-ring PAHs and four-ring PAHs were significantly higher in the morning period. All these differences show that the concentrations of gaseous PAHs both individually and according to the number of rings were higher in the morning period. This might be the result of mixing at a lower height, resulting in lower dispersion rates [34,35], or less efficient photolytic loss [36,37] in the morning period.

### 3.3. Comparison of Measurement Results with Other Studies

Since there has been little research investigating the concentration of gaseous PAHs in the ambient air of northern Thailand and U-SEA, it was necessary to compare the study’s findings to those of other urban cities around the world. Apart from Navarro et al. [38], who derived their gaseous PAHs from burning biomass, all of them were produced by vehicle emissions and fuel evaporation. Although a direct comparison of these results with those of other studies could not be accurate because of the difference in season, geographical location, sampling strategy, and analytical method, this comparison provides useful information for understanding trends in gaseous PAH concentrations in the environment. Table 2 presents data compiled for the relevant parameters of observed levels of gaseous PAHs across the globe. The findings of this study indicated that atmospheric gaseous PAHs in Chiang Mai were higher than those in Greece and the USA and lower than those in Vietnam, Taiwan, Japan, and China. Moreover, the studies in Table 2 revealed that gaseous PAHs in the ambient air of the Asia–Pacific region were found to be more abundant than those in other regions which may be attributable to the effects of local area and long-distance air masses being transported from neighboring countries. The results also showed the global variances in concentrations of the gaseous phase of individual PAHs. The ordering of the gaseous PAHs by quantity was found to be different. Meteorological conditions, the criteria used for gas pollutants, and photochemical oxidation are frequently blamed for differences in urban areas. For example, gaseous PAHs produced by primary emissions (i.e., vehicle emission and fuel evaporation) might be photo-oxidized and react with various high-potential oxidants (i.e., ozone, nitrate radical, and hydroxyl radical) in the atmosphere. Therefore, the difference in high traffic congestion could be the reason for the significant reactivity of those compounds because of widely detected gaseous PAH concentrations [7].

The prevalence of gaseous PAHs in Taiwan (Taichung), Japan (Shimizu and Fuji) and USA (California) showed the highest NAP concentrations in ambient air at 32.94%, 88.28%, 86.64%, and 85.99%, respectively. In our study, NAP was also the dominant compound, with a 51.86% ratio. However, it was discovered that the main order of gaseous PAHs followed the same pattern when compared to one case of biomass burning sources found in the USA (California), which was sampled near to the emission source. NAP, PHE, and FLU were the three most common chemicals, showing 87.61%, 5.77%, and 2.12%, respectively. The results for NAP, PHE, and FLU in Chiang Mai were 51.86%, 17.38%, and 9.32%, respectively. Furthermore, when the ring number of PAHs was compared, it was discovered that the order of biomass burning in the United States (California) was two-ring PAHs (87.61%), three-ring PAHs (10.88%), and four-ring PAHs (1.51%), whereas the order in this study was two-ring PAHs (52.07%), three-ring PAHs (38.30%), and four-ring PAHs (9.62%). In this study, as a result, during the sampling period at the receptor site, biomass burning was the main source of gaseous PAHs. These findings have never been published in either Thailand or upper Southeast Asia, which faced the same crisis at the same time. Particulate matter, such as PM_2.5_, is known to be a long-distance transport pollutant [39,40]. As a result, gaseous PAHs may be an important precursor of PAH derivatives adsorbed on PM_2.5_, potentially affecting human health.

**Table 2:** Comparison of gaseous PAHs with other countries.

City (Country)	Site	Period	Concentration (ng m^−3^) (Mean (SD))								Ref.
			NAP	ACY	ACE	FLU	PHE	ANT	FLA	PYR	
Chiang Mai, (Thailand)	Urban	Summer	61.52 (18.53)	4.15 (3.51)	6.86 (1.15)	10.96 (2.66)	20.44 (2.81)	2.84 (0.60)	5.57 (1.50)	5.80 (1.72)	This study
Athens (Greece)	Urban	Summer	-	-	-	1.28 (0.46)	6.08 (2.76)	0.89 (0.22)	2.79 (0.56)	1.91 (0.41)	[41]
Baltimore & northern Chesapeake Bay (USA)	Urban	Winter - Chesapeake	-	-	-	2.09	3.61	0.13	0.58	0.42	[42]
		Summer									
		- Chesapeake	-	-	-	2.65	5.57	0.18	0.848	0.548	
		- Baltimore inner harbor	-	-	-	4.03	12.5	0.312	3.43	2.14	
Guangzhou (China)	Urban	Summer (July)									[43]
		- Ground level (1.5 m)	-	2.73	0.23	3.67	35.92	4.5	34.02	32.97	
		- High level (25 m)		0.18	0.05	1.1	15.3	1.31	23.36	17.02	
		Spring (April)									
		- High level (25 m)	-	1.74	0.25	3.34	23.92	5.08	16.49	16.53	
Taichung (Taiwan)	Industry	Summer to Winter	409	177	196	129	90	158	80.5	79.9	[44]
	Urban		283	118	137	85.8	60	105	53.7	53.3	
	Rural		223	126	47.4	73.3	33.2	48.3	31.3	32.9	
Rome (Italy)	Urban	Winter	687 (580)	39 (18)	57 (20)	18 (8)	71 (22)	5.6 (1.9)	18 (9)	7.6 (6.0)	[45]
Shimizu (Japan)	Urban	Summer	174.29 (1.21)	-	3.54 (1.51)	5.56 (1.17)	17.25 (1.33)	0.32 (1.54)	1.85 (1.33)	1.51 (1.14)	[46]
		Winter	213.44 (1.17)	-	2.46 (1.34)	4.74 (1.10)	10.10 (1.14)	0.34 (1.44)	1.62 (1.16)	1.19 (1.30)	
Fuji (Japan)	Urban	Summer	213.01 (1.33)	-	6.42 (1.35)	9.84 (1.31)	26.27 (1.32)	0.42 (1.55)	4.57 (1.32)	3.00 (1.35)	
		Winter	345.00 (1.41)	-	2.87 (1.54)	5.77 (1.33)	12.57 (1.47)	0.93 (1.82)	3.20 (1.36)	2.86 (1.37)	
Heraklion (Greece)	Urban	Annual	-	-	-	5.2	19.8	3.3	4.7	6.3	[47]
Guangzhou (South, China)	Urban	Annual	2.1 (1.9)	3.9 (3.5)	0.8 (0.5)	22.0 (8.8)	196 (92)	29.8 (15.4)	35.4 (19.7)	21.2 (11.3)	[48]
Hanoi (Vietnam)	Urban	Summer	-	-	-	-	150 (54)	15 (6.1)	36 (14)	65 (30)	[49]
Delhi (India)	Urban	Winter	-	9.8	7.6	9.9	12	3.1	2.2	1.8	[50]
		Summer	-	2.6	1.9	2.8	4.9	1.2	0.8	0.6	
		Monsoon	-	1.2	0.8	3.8	1.5	0.5	0.6	0.4	
Akkalkuwa (India)	Rural	Winter	-	-	13.7 (4.0)	42.8 (15.8)	90 (35)	48.6 (26.2)	25.6 (12.8)	19.4 (8.5)	[51]
		Summer	-	-	1.3 (0.4)	3.8 (1.4)	49.8 (18.5)	7.7 (3.6)	3.4 (1.6)	0.8 (0.3)	
		Post-monsoon	-	-	0.7 (0.2)	2.3 (0.9)	35.3 (13.1)	9.4 (4.3)	2.4 (1.2)	0.6 (0.3)	
California (USA)	Prescribed fire firefighter	Training event	669 (7)	34 (9)	6 (4)	13 (6)	50 (7)	4 (6)	8 (6)	9 (6)	[38]
	Wildland firefighter	Willow Fire	3189 (3)	72 (4)	21 (4)	77 (4)	210 (3)	16 (5)	33 (3)	22 (5)	

### 3.4. Gas–Particle Partitioning of PAHs

The distribution of PAHs under field conditions is evaluated by using the particle-gas partition coefficient (*Kp*) according to the following equation [52].

*Kp* = *Cs*/*Cg*

where

*Kp*—gas–particle partitioning coefficient (m^3^ μg^−1^);
*Cs*—the measured particle-phase concentration (μg μg^−1^);
*Cg*—the measured gas-phase concentration (μg m^−3^).

Pongpiachan et al. [53] investigated gas-particle partitioning coefficients (*Kp*) in carbonaceous aerosols in Chiang Mai using the Dachs–Eisenreich model. The results from that study were also chosen to be used in this study because they were related to the same province, to estimate the particle-phase concentration during the sampling period of gaseous PAHs. As shown in Table 3, *Ms*/*Mg* ratios ranging from 10^−5^ to 10^−2^ were estimated. This finding could indicate that the distribution of individual PAHs in ambient air is significantly higher in the gaseous phase than in the particulate phase. As a result, the possibility of producing highly toxic PAH derivatives (i.e., nitro-PAHs) may have a significant effect on gaseous PAHs, which has not previously been published.

### 3.5. Variations in the Concentration of Gaseous-Phase PAHs

The concentration of gaseous PAHs in the atmosphere can be influenced by a variety of factors, including meteorological conditions and chemical oxidation reactions with oxidants. Temperature and humidity, for example, were discovered to have a strong relationship with PAH concentrations [44,54,55,56]. Li et al. [57], on the other hand, did not discover any significant correlation. Thus, it may be inferred that the relationship between the concentrations of PAHs and various other factors is extremely complex and situation-specific.

Due to the lack of information regarding the potential variation in PAHs among the gaseous PAHs in northern Thailand, several variables, including variations in the strength of the source, weather patterns, and chemical reactions with oxidants in the atmosphere, could alter the concentrations of gaseous PAHs. Furthermore, it should be noted that PAHs in particulate matter derived from a 24 h sampling period cannot accurately describe the factors that govern diurnal fluctuations. As a result, it is critical to collect such data using a short sampling time with gaseous PAHs, particularly in urban areas in Chiang Mai province, where air pollutants are known to be a mixture of source emissions with particulate matter (PM_2.5_).

### 3.6. Effect of Meteorological Conditions

As described in Table 4, temperature is the most important parameter that is usually mentioned as a primary controller in deposition/volatilization processes, which mix secondary sources of pollutants and atmospheric concentrations of persistent organic pollutants (POPs) [58,59]. In this study, a strong positive correlation (r = 0.675) was found between temperature and the concentration of four-ring PAHs. This could be because high temperatures changed the gas-particle partitioning of PAHs, promoting the volatilization of this group from the particle to the gaseous phase. Kong et al. [60] indicated that PAHs with a four-ring gaseous phase of PAHs were more often found in the summer than during the other seasons. Furthermore, temperature-driven evaporation from plants, soil, and road surfaces in urban areas has been proposed to explain the low molecular weight of PAHs [61,62]. However, Singla et al. [63] found that higher temperatures promote the faster degradation of PAHs, particularly those PAHs with a low molecular weight, which explains the strong negative relationship between temperature and two-ring PAHs (r = −0.502) and three-ring PAHs (r = −0.610). Relative humidity has a strong negative correlation with four-ring PAHs (r = −0.545). This is consistent with a previous study [55,56,64] that suggested that an increase in atmospheric humidity can enhance the binding of gaseous PAHs onto particles in the ambient air (such as PM_2.5_ and PM_10_), but a strong positive correlation with two-ring PAHs (r = 0.503) and three-ring PAHs (r = 0.622) was observed, implying that high humidity can increase the lifetime and the quantity mobilized by long-range transport from the emission source due to a reduction in photolytic loss [4,65,66].

Pressure was found to have a strong positive correlation (r = 0.641) with three-ring PAHs. This suggests that high pressure could actually reduce mobility and affect atmospheric stability conditions, resulting in an accumulation of this group at the sampling site [67], whereas a four-ring PAH had a strongly negative correlation to ambient pressure (r = −0.797), implying that increased atmospheric pressure could improve PAH binding to the particles in the ambient air [55].

### 3.7. Effect of Gaseous Pollutants

It should also be noted that a lack of correlation in meteorological conditions does not always imply a lack of dependence. However, other factors, such as interaction with various oxidants in the ambient air, may have a significant impact [68].

Apart from ANT, all compounds have a weaker or no correlation with NOx, implying that their concentrations are governed by different primary sources and/or processes. However, NOx has a moderately positive correlation with temperature (r = 0.499), suggesting that the relationship between PAHs and NOx may be influenced by the magnitude of primary or secondary input rather than the atmospheric boundary layer (ABL) [61].

Ozone was also found to have a strong positive correlation with four-ring PAHs, implying that the increase in four-ring PAHs in the atmosphere could be due to photochemical oxidation reactions induced by solar irradiation [69]. Moreover, the majority of gaseous PAHs appeared to have no correlation with a group of pollutants (i.e., PM_10_, CO, NO_2_, and SO_2_), implying that they were not derived from the same sources and did not share the same transport pattern [47,70]. Because those pollutants are released from traffic congestion [71], it is possible to conclude that the majority of the gaseous PAHs identified are not produced solely as a result of traffic congestion.

Although Arey et al. [72] found that the rate of PAH degradation increases in urban areas when oxidants are presented, which might suggest a strong correlation with gaseous PAHs, this study found only weak correlations between gaseous PAHs and ozone, sulfur dioxide, and carbon monoxide, as shown in Table 5. These substances might be capable of interacting with stronger oxidants like hydroxy radicals (OH•), nitrate radicals (NO_3_•), and ozone (O_3_) [73,74,75,76,77,78,79]. According to Keyte et al. [80], most three- to four-ring PAHs appear to react with OH• at rates of up to five orders of magnitude higher than those of reactions with NO_3_•. However, ozone reactions in the atmosphere seem to be insignificant. Thus, it is reasonable to assume that most PAH derivatives adsorbed on particles in the ambient air, such as PM_2.5_, may be produced through a reaction with OH• and NO_3_•. Numerous studies have been conducted to assess the product yield of these reactions, including those on NAP [81], ACE [82], ACY [82], FLU [83], PHE [82], and ANT [73], as shown in Table 6. As a result, in this study, gaseous PAHs were used to estimate the possibility of producing nitro-derivatives by percentage yield obtained with oxidants (OH• and NO_3_•). For example, NAP could be transformed to 1-Nitronaphthalene by about 0.3% by OH• which means the NAP 61.52 ng m^−3^ detected could be changed to 0.18 ng m^−3^. Therefore, it can be concluded that the nitro-PAH yields obtained ranged from 0.02 ng m^−3^ (8-Nitrofluoranthene) to 10.46 ng m^−3^ (1-Nitronaphthalene).

Based on published scientific studies, the IARC Working Group on the Evaluation of Carcinogenic Risks to Humans classified several nitro-PAHs as having a higher potential for carcinogenic compounds than their parents, including 5-nitroacenaphthene, 4-nitroacenaphthylene, 2-nitrofluorene, and 3-nitrofluorene. As a result, this study found that gaseous PAHs produced various nitro-derivatives in the ambient air which accumulated on particulate phases such as PM_2.5_. Therefore, during a smoke haze period, it is possible that local people might be exposed to hazardous chemicals such as nitro-derivatives which accumulate in the body every year. This has not been previously reported.

### 3.8. Determination of PAH Emission Sources

A correlation analysis was used to determine the relationships between individual PAHs and their possible sources, with the assumption that two or more compounds may correlate due to a similar origin or atmospheric behavior. Diesel fuel, for example, has been found to have a high impact on low-molecular-weight (LMW) compounds, whereas high-molecular-weight (HMW) compounds are typically present near detection limits [84,85,86]. HMW compounds, on the other hand, have higher emission rates during diesel fuel combustion than LMW PAH, which is attributed to their pyro-synthesis during fuel combustion in engines [84,85,87]. Several methods were used in this study to investigate the potential role of gaseous PAHs as a good source tracer, which have not previously been used to identify the source of air pollution in this area.

#### 3.8.1. Pearson Correlation

According to de Rocha et al. [88], Pearson correlation coefficients are shown in Table 7. A significant correlation between FLA and PYR was discovered (r = 0.768), which might suggest that the emission sources are either diesel or gasoline. Additionally, the PHE, FLT, and PYR moderate-to-strong correlations (0.500 < r < 1.000) suggest that the source of these compounds may be diesel exhaust from heavy-duty vehicles. Additionally, it was discovered that, except for NAP and ANT (r = 0.626) and PHE and ACE (r = 0.565), there was a weak-to-moderate correlation between NAP, ACY, ACE, FLU, PHE, and ANT (0.00 < r < 0.499). This could be tentatively attributed to wood combustion for domestic heating/energy production and emissions from a petroleum refinery. Since more than one or two sources could be involved in the origin of each PAH, it is possible that the more diverse sources of PAHs in this sampling site would reflect more complicated correlations among other compounds. Finally, it can be said that multiple sources contributed to the production of gaseous PAHs in this region.

#### 3.8.2. Diagnostic Ratios of PAHs

During the sampling period, the diagnostic ratios were also calculated to identify the potential sources of PAHs. According to Tobiszewski and Namienik [89], although all phases of PAHs can be examined using diagnostic ratios, particulate-phase PAHs were studied more frequently. It is possible that the relatively simple particle-phase sampling procedure, as well as the determination of gaseous PAHs, were rather complicated, involving chemical and physical properties, reactions with oxidants, and solar radiation, all of which can change the sample’s fingerprint by changing the true source results [90]. However, short-term sampling durations of gaseous PAHs could reduce these uncertainties [19]. As a result, the findings of this study can be used to demonstrate the potential of gaseous PAHs in the identification of the sources of PAHs.

From the samples examined in this study, two specific PAH ratios were computed. The method for identifying the source of PAHs during the sampling period involved using a scatter plot of the pair ratios of ANT/(ANT + PHE) and FLA/(FLA +PYR). Reference values for identifying sources were provided by various other studies [51,91,92]. When FLA/(FLA + PYR) was less than 0.40, for instance, it was likely to have come from petroleum; however, when these values were greater than 0.50, it was likely to have come from burning biomass or coal. The source of the ratios, which ranged from 0.40 to 0.50, was determined to be the combustion of fossil fuels. The Ant/(ANT + PHE) ratios can also be used to illustrate the distinction between pyrogenic and petrogenic sources by using a reference point of 0.1 [57,92].

**Figure 5:**
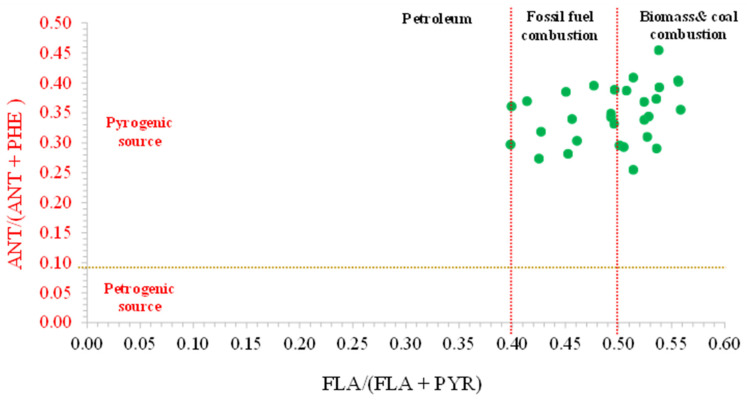
Cross plot of the diagnostic ratios for the sources identification of gaseous PAHs in sampling period (green circles = correlation of ratios of FLA/(FLA+PYR) in X-axis and ratios of ANT/(ANT + PHE) in Y-axis) for 30 samples during the sampling period; red lines color = critical ratios of FLA/(FLA+PYR) for source separation; yellow color = critical ratio of ANT/(ANT + PHE)).

Figure 5 shows that, in this study, the PAH ratios of FLA/(FLA + PYR) ranged from 0.40 to 0.56, indicating that biomass, coal, and petroleum combustion all played a role. Furthermore, the ANT/(ANT + PHE) ratio varied from 0.25 to 0.45, indicating a significant contribution from pyrogenic sources such as the incomplete combustion of fossil fuels and organic matter.

In conclusion, our investigation identified a mixed contribution from petroleum, biomass, and coal combustion as the main sources of PAHs in Chiang Mai during the smoke haze period. These results correspond to the topography dimensions and human activities during the sampling period. Chiang Mai is surrounded by mountains, and its geography resembles a bowl, and different kinds of air pollution may be produced because of urbanization. Therefore, anthropogenic gaseous PAHs in local areas could be produced from various local sources (i.e., forest fires, the burning of agricultural residues, and vehicle emissions). Moreover, biomass burning activities from neighboring provinces and neighboring countries during the sampling period could also have impacted the accumulation of those compounds owing to long-distance transport.

#### 3.8.3. Principal Component Analysis (PCA)

Multivariate statistical principal component analysis (PCA) was employed to examine the influence of various types of emissions on PAH concentrations and distribution [93,94,95]. In this study, the outcomes of PCA were combined with diagnostic ratios as has been extensively utilized by numerous publications for the preliminary discrimination of PAH sources in urban areas [96,97,98]. Particulate-phase PAHs have been examined and reported on in earlier studies in terms of the composition of both qualitative and quantitative data. They were discovered to be impacted by variations in meteorological conditions: local circulation, long-range atmospheric transport, and emission strength. Consequently, various key findings from these investigations were obtained in order to determine the emission sources. For example, Yang et al. [94] indicated that industrial emissions were linked with high levels of four-ring and five-ring PAHs (BaA and BaP), particularly from heavy-duty diesel vehicles (based on BbF). Moreover, Wang et al. [99] discovered four different sources of particle-bound PAH compounds in PM_2.5_ and PM_10_: (i) industry (BPER, BbF, BkF, DbA, CHR, and ACE), (ii) coal combustion (FLU, PHE, ANT, FLA, PYR, BaA, and CHR), (iii) traffic emissions (BkF, BbF, BaA, CHR, and BPER), and (iv) biomass combustion (NAP, ACY, ACE, FLU, BaA, and DbA).

Even though gaseous PAHs were produced in greater quantities from a variety of sources and showed more source-specific characteristics than particle-phase PAHs, they received less attention [16,99,100]. To investigate potential emission sources, the gaseous PAHs detected in this research were compared to particulate-phase PAHs in Chiang Mai from earlier studies. Table 8 shows that the three principal components, PC1, PC2, and PC3, represented 34.2%, 22.9%, and 16.0%, respectively, of the total variance of the data during the sampling period.

PC1 contributed 34.2% of the data variance, due to the heavy loading of NAP, ANT, and FLU. According to earlier research, NAP is the primary PAH in coal combustion or biomass burning while FLU and ANT are characteristic of wood combustion or mass combustion [38,101,102,103,104]. Additionally, coal and biomass combustion at low-to-moderate temperatures, as well as petrogenic sources, showed a high significant correlation with NAP and FLU [105]. ANT has been identified as a marker for wood combustion [106,107]. Liu et al. [108] established that NAP was created from petrogenic and pyrogenic sources. Overall, PC1 was chosen to represent the combined sources (i.e., sources from vehicles, coal combustion, and biomass burning).

**Table 8:** Factor loading of gaseous PAHs in the PCA model.

Gaseous PAHs	Principal Component		
	PC-1	PC-2	PC-3
NAP	***0.619***	0.366	−0.140
ANT	***0.556***	0.498	−0.534
PHE	0.156	***0.861***	−0.050
FLU	***0.702***	0.185	−0.032
ACE	0.144	***0.802***	0.378
ACY	0.141	0.168	***0.892***
FLA	−0.842	−0.057	−0.161
PYR	−0.882	−0.073	−0.118
Variance (%)	34.210	22.973	16.070
Cumulative %	34.210	57.183	73.254
Suggested sources	Multiple sources (Biomass burning, coal combustion and vehicle emission)		Biomass burning

Remark: The gaseous PAHs are more susceptible to reaction than in the particulate phase [20]; therefore, the high correlation of variables should be set greater than 0.500 [108], as shown in Italicized Letters in Bold.

PC-2 contributed 22.9% to the total variance; it was highly loaded with PHE and ACE. These chemicals are mostly produced at a low combustion temperature (as in coal and biomass combustion) [109,110]. Furthermore, ACE is an indicator of residential wood burning [103,111,112]. This component is highly compatible with the typical emission sources from wood burning. PHE, on the other hand, was shown to predominate in coal combustion [113,114] and vehicle emissions [104], revealing that PHE is the most prevalent PAH species in diesel emissions [63,109]. Moreover, it is produced by the burning of fuel at a low temperature [108]. As a result, PC-2 was designated as the source of coal combustion, biomass burning, and automobile emissions.

PC-3 provided 16.0% of the total variance, with ACY being the only high loading PAH. According to Fang et al. [111] and Kamal et al. [112], ACY is also recognized as a tracer for residential wood combustion. This component is quite consistent with the typical emission sources from wood burning. Therefore, PC-3 was selected as an emission source of biomass burning.

As a result, it can be concluded that the greatest variances based on PC-1 of gaseous PAHs were generated via a mixture of coal combustion, biomass burning, and vehicle emissions, with similar results to a previous study of particulate matter [30].

Ultimately, the combination of diagnostic ratios and PCA indicate that during the sampling period, mixed sources including coal combustion, biomass burning, and vehicle emissions are represented. It should be emphasized that the diagnostic ratios and PCA model were unable to distinguish between regional and local emissions. However, the impacts of PAHs drifting from the surrounding cities in Chiang Mai province should not be ignored.

The results of source identification can be supported by the low correlation of gaseous PAHs and the groups of pollutants discussed above (i.e., PM_10_, CO, NO_2_, and SO_2_), which are transported in different patterns implying that most of the gaseous PAHs detected were not generated solely by traffic congestion.

## 4. Conclusions

The concentrations of gaseous PAHs in the ambient air were measured in a smoke haze period in the morning and afternoon periods. It was found that the levels of most gaseous PAHs were found to be higher in the morning period. The total concentrations of eight gaseous PAHs were 125 ± 22 ng m^−3^ (morning period) and 111 ± 21 ng m^−3^ (afternoon period). Five compounds, including ACE, FLU, PHE, FLA, and PYR, showed significant differences between the two sampling periods when comparing individual compounds, while three-ring and four-ring PAHs were significantly different when comparing according to the number of rings. Then, the correlation between the gaseous PAHs and both atmospheric meteorological conditions and other pollutants were further calculated. It showed that meteorological conditions including relative humidity, temperature, and pressure could affect, in descending order, two-ring, three-ring, and four-ring PAHs. However, all compounds have a weak or no correlation with other pollutants, except four-ring PAHs and O_3_, which have a strong positive correlation, implying that the increase in O_3_ in the ambient air may have affected the degradation of four-ring PAHs in the atmosphere due to photochemical oxidation reactions induced by solar irradiation.

Source identification was also determined based on the Pearson correlation, diagnostic ratios, and PCA. It was found that gaseous PAHs were mostly generated from mixed sources (biomass burning, coal combustion, and traffic emission). These findings followed the same trends as previous research on PAH-bound particles (i.e., PM_2.5_) in northern Thailand. Therefore, this research indicates that gaseous PAHs have high potential for source identification in northern Thailand and U-SEA. Furthermore, the detected gaseous PAHs could be used to calculate the ratio of gaseous to particulate phases of individual PAHs in ambient air, as well as PAH derivatives, which are highly toxic for human health.

## Figures and Tables

**Table 1:** Concentration of gaseous PAHs detected during different two sampling periods.

Compound	Number of Ring	Concentration (ng m^−3^)			
		Individual Compound		Ring Compound	
		Min–Max	Average ± SD	Min–Max	Average ± SD
NAP	2	31–94	62 ± 19	31–94	62 ± 19
ACY	3	0.88–14	4.1 ± 3.5	33–60	45 ± 6.3
ACE		4.9–8.9	6.9 ± 1.1		
FLU		7.9–18	11 ± 2.7		
PHE		15–27	20 ± 2.8		
ANT		2.0–4.1	2.8 ± 0.6		
FLA	4	3.5–9.2	5.6 ± 1.5	6.5–18	11 ± 3.0
PYR		3.0–9.0	5.8 ± 1.7		

**Table 3:** Particle-phase concentration estimated by gas-particle partitioning coefficient.

Compound	^a^ *K_p_* (m^3^ μg^−1^)	^b^ *C_g_* (μg m^−3^)	*C_s_* (μg μg^−1^)	Distribution Ratio of *Ms*/*Mg*
NAP	-	6.15 × 10^−3^	-	-
ACY	1.96 × 10^−5^	4.15 × 10^−3^	8.13 × 10^−8^	1.96 × 10^−5^
ACE	2.76 × 10^−5^	6.86 × 10^−3^	1.89 × 10^−7^	2.76 × 10^−5^
Flu	6.16 × 10^−5^	1.10 × 10^−3^	6.78 × 10^−8^	6.16 × 10^−5^
HE	3.13 × 10^−4^	2.04 × 10^−3^	6.39 × 10^−7^	3.13 × 10^−4^
ANT	3.39 × 10^−4^	2.84 × 10^−3^	9.63 × 10^−7^	3.39 × 10^−4^
FLA	3.25 × 10^−3^	5.57 × 10^−3^	1.81 × 10^−5^	3.25 × 10^−3^
PYR	3.01 × 10^−2^	5.80 × 10^−3^	1.75 × 10^−4^	3.01 × 10^−2^

Remark: ^a^ [52]; ^b^ results for this study; *Ms* = distribution of each PAH in the particulate phase (μg), and *Mg* = distribution of each PAH in the particulate phase (μg).

**Table 4:** Pearson correlation coefficients between gaseous PAHs and meteorological conditions in the ambient air during the sampling period over Chiang Mai, Thailand.

	Wind Speed	Net Radiation	Temperature	Pressure	Relative Humidity
NAP	0.002	−0.339	***−0.502*** **	0.414 *	***0.503*** **
ACY	−0.202	−0.059	0.024	0.180	−0.065
ACE	−0.106	***−0.679*** **	***−0.630*** **	0.492 **	***0.680*** **
ANT	0.090	−0.186	***−0.559*** **	0.491 **	***0.572*** **
PHE	−0.147	−0.489 **	***−0.523*** **	0.427 *	***0.594*** **
FLU	−0.325	−0.291	−0.449 *	***0.596*** **	0.329
FLA	0.101	0.416 *	***0.608*** **	***−0.676*** **	−0.493 **
PYR	−0.022	0.331	***0.659*** **	***−0.814*** **	***−0.530*** **
2-ring PAHs	0.002	−0.339	***−0.502*** **	0.414 *	***0.503*** **
3-ring PAHs	−0.19	−0.479 **	***−0.610*** **	***0.641*** **	***0.622*** **
4-ring PAHs	0.037	0.394 *	***0.675*** **	***−0.797*** **	***−0.545*** **
8-PAHs	−0.047	−0.369 *	***−0.506*** **	0.424 *	***0.528*** **

Remark: * Correlation is significant at the 0.05 level (2-tailed). ** Correlation is significant at the 0.01 level (2-tailed), and the high correlation of variables should be set to greater than 0.500, as displayed in Italicized Letters in Bold.

**Table 5:** Pearson correlation coefficients between gaseous PAHs and other pollutants in the ambient air during the sampling period over Chiang Mai province.

	NO_2_	NO	NO_X_	CO	SO_2_	O_3_	PM_2.5_	PM_10_
NAP	−0.272	−0.165	−0.232	0.072	0.225	−0.385 *	0.215	−0.315
ACY	0.125	0.291	0.221	0.213	0.063	−0.062	−0.098	−0.237
ACE	−0.162	−0.119	−0.156	0.367 *	−0.075	***−0.568*** **	−0.121	−0.311
ANT	***−0.541*** **	−0.366 *	***−0.501*** **	0.079	−0.064	−0.289	−0.263	−0.418 *
PHE	−0.231	−0.203	−0.246	0.236	−0.070	−0.434 *	−0.158	−0.420 *
FLU	−0.026	0.194	0.079	0.168	0.130	−0.437 *	−0.076	−0.115
FLA	0.262	0.027	0.188	−0.396 *	−0.024	***0.559*** **	0.278	0.26
PYR	***0.559*** **	0.189	0.445 *	−0.195	0.105	0.440 *	0.34	***0.526*** **
2-ring PAHs	−0.272	−0.165	−0.232	0.072	0.225	−0.385 *	0.215	−0.315
3-ring PAHs	−0.292	−0.086	−0.218	0.338	−0.024	−0.493 **	−0.259	***−0.569*** **
4-ring PAHs	0.447 *	0.121	0.346	−0.307	0.047	***0.526*** **	0.332	0.427 *
8-PAHs	−0.252	−0.147	−0.211	0.116	0.189	−0.394 *	0.157	−0.366

Remark: * Correlation is significant at the 0.05 level (2-tailed). ** Correlation is significant at the 0.01 level (2-tailed), and the high correlation of variables should be set to greater than 0.500, as displayed in Italicized Letters in Bold.

**Table 6:** Estimation of the yield of nitroarene products generated by gas-phase reactions of polycyclic aromatic hydrocarbons known to be present in ambient air with hydroxyl radicals and nitrate radicals (both in the presence of NO_3_•).

Parent PAHs (1° PAHs)		PAH Derivatives (2° PAHs)		
Name	Detected Concentration ^a^ (ng m^−3^)	Name	Obtained Yield (ng m^−3^) ^b^ from 1° PAHs Reacted with	
			OH•	NO_3_•
NAP	61.52	1-Nitronaphthalene	0.1846	10.4584
		2-Nitronaphthalene	0.1846	4.3064
ACY	6.86	5-nitroacenaphthene	0.0137	0.1029
		4-nitroacenaphthene	0.0137	2.7440
		3-nitroacenaphthene	0.0137	0.1372
ACE	4.15	4-Nitroacenaphthylene	0.0830	-
FLU	10.96	3-nitrofluorene	0.1534	-
		1-nitrofluorene	10.9600	-
		4-nitrofluorene	0.0329	-
		2-nitrofluorene	0.0110	-
PHE	20.44	2 nitroisomers (Not 9-nitrophenanthrene)	-	-
		4 nitroisomers (Including 9-nitrophenanthrene)	-	-
ANT	2.87	1-Nitroanthracene	-	-
		2-Nitroanthracene	-	-
FLA	5.57	2-Nitrofluoranthene	0.0057	-
		7-Nitrofluoranthene	0.0057	-
		8-Nitrofluoranthene	0.1671	1.3368
PYR	5.8	2-nitropyrene	0.0557	-
		4-nitropyrene	0.0167	-

Remark: ^a^ detected PAHs from this study and ^b^ obtained yield calculated from % yield of reaction pathway in ambient air [74].

**Table 7:** Pearson correlation coefficients between individual PAHs in this study.

	NAP	ACY	ACE	ANT	PHE	FLU	FLA	PYR	2-Ring PAHs	3-Ring PAHs	4-Ring PAHs	8- PAHs
NAP	1.000											
ACY	0.351	1.000										
ACE	0.247	0.082	1.000									
ANT	***0.626*** **	0.005	0.290	1.000								
PHE	0.287	−0.225	***0.565*** **	0.432 *	1.000							
FLU	0.457 *	0.080	0.202	0.405 *	0.297	1.000						
FLA	−0.355	−0.230	−0.287	−0.347	−0.260	−0.470 **	1.000					
PYR	−0.424 *	−0.246	−0.261	−0.485 **	−0.238	−0.467 **	0.768 **	1.000				
2-ring PAHs	1.000 **	0.351	0.247	***0.626*** **	0.287	0.457 *	−0.355	−0.424 *	1.000			
3-ring PAHs	0.673 **	0.480 **	***0.619*** **	***0.705*** **	***0.632*** **	0.478 **	−0.485 **	***−0.537*** **	***0.673*** **	1.000		
4-ring PAHs	−0.417 *	−0.253	−0.290	−0.447 *	−0.264	−0.498 **	0.932 **	***0.948*** **	−0.417 *	***−0.545*** **	1.000	
8-PAHs	***0.980*** **	0.399 *	0.346	***0.670*** **	0.387 *	0.455 *	−0.311	−0.381 *	***0.980*** **	***0.780*** **	−0.370 *	1.000

Remark: * Correlation is significant at the 0.05 level (2-tailed). ** Correlation is significant at the 0.01 level (2-tailed), and the high correlation of variables should be set to greater than 0.500, as displayed in Italicized Letters in Bold.

## Data Availability

Data are contained within the article.

## Supplementary Materials

The following supporting information can be downloaded at: https://www.mdpi.com/article/10.3390/toxics11120990/s1, Table S1: The recoveries of PAHs from the spiking method and SRM-1649 urban dust sample; Table S2: Analytical characteristics of gaseous PAHs analyzed by GC–MS; Table S3. Method detection limit of developed gaseous PAHs sampling device based on biochar and XAD-2 as adsorbent.
