# Supplementary material for: Impact of Atmospheric Conditions and Source Identification of Gaseous Polycyclic Aromatic Hydrocarbons (PAHs) during a Smoke Haze Period in Upper Southeast Asia
Source: Toxics. 2023 Dec 5;11(12):990. doi: 10.3390/toxics11120990 (PMC10748124; doi:10.3390/toxics11120990)
Supplement: Supplementary file 1 [file toxics-11-00990-s001.zip › toxics-2735331-supplementary.pdf]

Supplementary materials

Table S1. The recoveries of PAHs from the spiking method and SRM-1649 urban dust sample.

Compound	% Recovery of sample treatment (Mean ± SD)	
	Spiking method	SRM 1649b
	Extraction procedure with SPE clean-up procedure	Extraction procedure with SPE clean-up procedure
NAP	81 ± 1	64 ± 2
ACY	92 ± 3	98 ± 3
ACE	93 ± 9	95 ± 5
FLU	92 ± 5	98 ± 9
PHE	89 ± 3	85 ± 3
ANT	96 ± 2	92 ± 6
FLU	94 ± 4	91 ± 5
PYR	91 ± 1	84 ± 2

Table S2. Analytical characteristics of gaseous PAHs analyzed by GC–MS.

Compound	Detection limit (µg mL^{-1})		Repeatability (% RSD)		Reproducibility (%RSD)		Concentration Linearity (ng mL^{-1})
	LOD	LOQ	Low range (6 ng mL^{-1})	High range (60 ng mL^{-1})	Low range (6 ng mL^{-1})	High range (60 ng mL^{-1})	
NAP	0.053	0.177	0.775	0.560	2.366	3.678	1-3,000
ACY	0.039	0.129	0.589	0.462	0.901	3.419	1-3,000
ACE	0.034	0.114	0.847	0.669	1.000	3.371	1-3,000
FLU	0.040	0.132	0.571	0.700	1.006	3.238	1-3,000
PHE	0.032	0.107	0.575	0.703	0.685	3.366	1-3,000
ANT	0.040	0.132	0.944	0.964	0.904	3.244	1-3,000
FLU	0.036	0.121	0.702	2.574	1.286	3.249	1-3,000
PYR	0.038	0.126	1.477	3.488	5.003	2.351	1-3,000

Table S3. Method detection limit of developed gaseous PAHs sampling device based on biochar and XAD-2 as adsorbent.

Compounds	Method detection limit (ng m^{-3})			
	Biochar		XAD-2	
	LOD	LOQ	LOD	LOQ
NAP	4.43	14.76	1.69	5.63
ACY	0.26	0.86	0.29	0.96
ACE	0.74	2.48	1.07	3.57
FLU	0.38	1.28	0.44	1.46
PHE	0.46	1.55	0.71	2.36
ANT	1.06	3.54	0.79	2.62
FLU	0.31	1.04	0.72	2.40
PYR	0.49	1.62	0.97	3.23
